# Could photoperiodic responses have evolved before the emergence of circadian clocks?

**DOI:** 10.1111/nph.70598

**Published:** 2025-10-01

**Authors:** Maria Luísa Jabbur, Carl Hirschie Johnson

**Affiliations:** ^1^ John Innes Centre, Norwich Research Park Colney Ln Norwich NR4 7UH UK; ^2^ Vanderbilt University 2201 West End Ave Nashville TN 37235 USA

**Keywords:** circadian rhythms, cyanobacteria, evolution, evolution of photoperiodic responses, photoperiodism

## Abstract

Plants use photoperiod (i.e. day length) as a seasonal cue for timing when to flower. This ability, known as photoperiodism, also underlies phenomena such as migration, seasonal reproduction, and hibernation in animals. Because a circadian (daily) clock underlies the day/night length timing mechanism in most organisms, it has been generally assumed that circadian rhythms evolved before the ability to measure the photoperiod. Our recent discovery that adaptive photoperiodic responses extend as far back as bacteria, with cyanobacteria showing a photoperiodic response remarkably similar to those of eukaryotes, has led us to question this assumption. In this Tansley insight, we put forward a new hypothesis for how photoperiodism might have evolved which is based on the evolutionary implications of bacteria being capable of photoperiodic responses.

## Resumo

As plantas utilizam o fotoperíodo (isto é, a duração do dia e da noite) como uma pista sazonal para saberem quando devem florir. Esta habilidade, conhecida como fotoperiodismo, também está por trás de outros fenômenos sazonais tais como migracão, reprodução sazonal e hibernação. Dado o fato de que relógios circadianos estão envolvidos no mecanismo de medição do dia e da noite na maior parte dos organismos estudados, geralmente se supõe que os ritmos circadianos evoluíram antes da capacidade de medir o fotoperíodo. A nossa descoberta recente de que bactérias também possuem respostas fotoperiódicas semelhantes a de eucariotos nos faz questionar essa suposição. Neste Tansley insight, nós postulamos uma nova hipótese a respeito de como o fotoperiodismo pode ter evoluído, nos baseando nas implicações evolutivas da presença do fotoperiodismo em bactérias.

**Table:** 

	Contents	
	Summary	2707
I.	Cyanobacteria: rhythm in blue(‐greens)	2707
II.	Beyond circadian rhythms	2707
III.	A clock for all seasons, or did all seasons make a clock?	2708
	Acknowledgements	2711
	References	2712

## I. Cyanobacteria: rhythm in blue(‐greens)

Cyanobacteria are the oldest lineage that is still in existence. While our planet is estimated to be *c*. 4.54 billion years old (Bya), cyanobacteria have inhabited it for over half (potentially 75%) of that time (Sánchez‐Baracaldo *et al*., 2022). They are an incredibly diverse group that occupies a wide array of ecological spaces, such as aquatic environments, soil, and the gut microbiome, as well as in association with organisms like plants, sponges, and corals (Whitton, 2012). They have been crucial in creating the global environment we have today, in part by playing a role in the oxygenation of our planet – allowing for life to become increasingly complex and diverse – but also by giving rise to chloroplasts, *c*. 1.9 billion years ago (Sánchez‐Baracaldo *et al*., 2017).

This widespread geographical and ecological distribution, important biogeochemical role, and ancient evolutionary history make cyanobacteria an important model organism across diverse fields. One of these fields is that of chronobiology (Box 1), where despite the infancy of the cyanobacterial circadian field (relative to plants (1729–1825, Sweeney, 2013), fungi (Pittendrigh *et al*., 1959), and mammals (Ritchter, 1922)), cyanobacteria now have the most well‐described circadian clock, with atomic level resolution of its inner workings.

Box 1 **Circadian rhythms**

Usually, if one thinks about chronobiology and cyanobacteria, one is often thinking about the realm of circadian clocks. These clocks, which are pervasive across the tree of life (Larrondo, 2025), coordinate a myriad of internal rhythms, and can be described as self‐sustained oscillators which: (1) can maintain a *c*. 24 h rhythmicity even in the absence of external stimuli; (2) can be entrained by environmental cycles such as the light : dark cycle; and (3) are temperature compensated, meaning that their periodicity remains close to 24 h when faced with temperature changes, within a permissible range (Dunlap *et al*., 2004). First described in plants in the 18^th^ century, it was not until the late 20^th^ century that cyanobacteria (or any bacterium) were discovered to possess circadian clocks (Grobbelaar *et al*., 1986). Since then, their circadian rhythms have been minutely studied, and their oscillator was found to be composed of only three proteins: KaiA, KaiB, and KaiC (Ishiura *et al*., 1998), with the main mechanism of the clock being a *c*. 24 h phosphorylation cycle of KaiC that regulates the rhythmic association/dissociation of a KaiA/KaiB/KaiC complex. Of note, the three clock genes of cyanobacteria are evolutionarily ancient – kaiC predates crown Cyanobacteria at 3.8–3.5 billion years old (Bya), while kaiB and kaiA evolved closer to the evolution of cyanobacteria, between 3–2.6 Bya and 2.9–2.6 Bya, respectively (Dvornyk *et al*., 2003; Dvornyk & Mei, 2021; Jabbur & Johnson, 2022).

## II. Beyond circadian rhythms

**Fig. 1:**
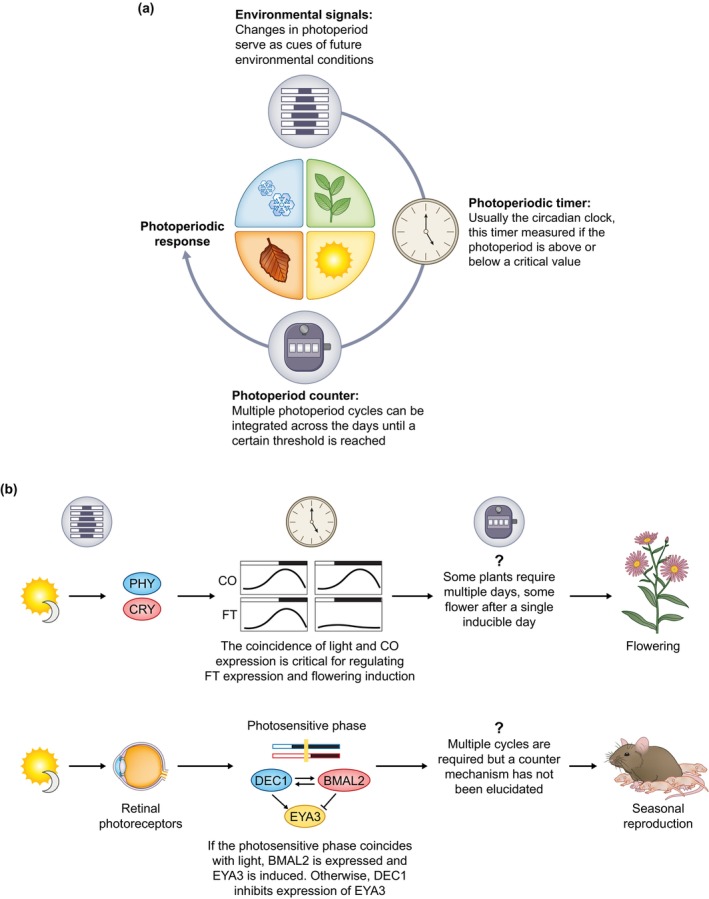
(a) Model for how photoperiodic time measurement works across different species. Changes in photoperiod work as the main environmental signal of upcoming seasons. The length of the photoperiod is measured by a photoperiodic timer, which is often a circadian clock. Multiple photoperiodic cycles are integrated by a photoperiodic counter, and after a certain amount of cycles is reached, the photoperiodic response is generated. (b) Examples of the photoperiodic pathways in plants (upper) and mammals (lower).

Yet, there is more to the field of chronobiology than simply circadian rhythms. For over 100 years (Garner & Allard, 1920; Marcovitch, 1924), it has been known that organisms not only keep track of the daily day–night cycles, but also of the annual cycles of day/night length (i.e. photoperiod; Box 2). Similarly to circadian clocks, there is limited homology across phylogenetic groups when it comes to the genes that underlie photoperiodic responses: within kingdoms, we see homology of certain genes (Serrano‐Bueno *et al*., 2017; Abrieux *et al*., 2020), but only the CRY photoreceptors and the kinase CK1 appear to show homology *across* kingdoms. However, there is remarkable conservation when it comes to the general mechanisms underlying photoperiodic responses: photoperiodic signals are thought to be measured by a ‘photoperiodic timer’, which in most organisms appears to be a circadian clock. The signals are integrated across multiple days through a ‘photoperiodic counter’; day lengths above (for long‐day responses) or below (short‐day) a certain threshold induce the accumulation of an inducer. Once this inducer is accumulated to high enough levels, it can trigger the switch between short‐day and long‐day phenotypes (e.g. vegetative to flowering stages) by acting on a series of downstream effectors (see Fig. 1a for a general diagram, and 1b for specific examples from plants and mammals).

Box 2 **Photoperiodism**

The annual cycle of day/night‐length is often a reliable cue of upcoming environmental conditions: due to the thermal capacity of our planet, changes in day length often precede changes in temperature (Dodds *et al*., 2016). The shortest day of the year happens *c*. 1 (for air, Hut *et al*., 2013) to 2 (water, Jabbur *et al*., 2021) months before the coldest day, and similarly for the longest and hottest days. Temperature is a major stressor for many organisms, and the yearly cycle of ambient temperature can thus create annually recurring windows of stress and opportunity, which can be predicted through the very reliable cue of day length. It is therefore not surprising that many organisms have evolved the ability to use photoperiods as a cue of upcoming seasons, and change their metabolism, physiology, and behavior when presented with particular photoperiods, generating incredibly diverse annual responses like flowering, migration, seasonal reproduction, and hibernation (Nelson *et al*., 2010).

Much of the research in the field of photoperiodism has focused on the mechanistic basis of this phenomenon – with great progress being made in plants, birds, insects, and mammals – or on how photoperiodism evolves in a context of latitudinal clines. Less focus has been devoted to the origins of photoperiodism, and when and how it might have first evolved. In this context, our recent discovery that photoperiodism exists in bacteria (Jabbur *et al*., 2024) is particularly consequential. Up until recently, photoperiodism was thought to be an exclusively eukaryotic phenomenon, but as we have demonstrated, cyanobacteria of the species *Synechococcus elongatus* are capable of photoperiodic time‐measurement (PPTM) in a way that is remarkably like eukaryotes (Fig. 1). In our studies, we have tested the cold sensitivity of cells that had been exposed to different photoperiods, simulating winter, spring/fall, and summer. Cells grown at constant 30°C that were exposed to short, winter‐like days could survive cold exposure two to three times better than those exposed to long, summer‐like days. Remarkably, this response necessitated the circadian clock (likely the photoperiodic time‐measurer), and only occurred after multiple (4–6) days of exposure to short photoperiods, indicating that the cells possess a counter mechanism that is integrating the photoperiodic information across the days and generations. Once this information is integrated, it appears to drive preemptive changes in lipid membrane saturation levels, mimicking those observed after a 10°C step‐down in temperature.

## III. A clock for all seasons, or did all seasons make a clock?

The fact that organisms as ancient as cyanobacteria can show photoperiodic responses suggests that this ability is evolutionarily more ancient than previously appreciated. At the moment, we are limited by the fact that we have only studied this response in one bacterial species, and that the mechanism behind it is not yet known. While future studies will help to clarify, this observation nevertheless opens up a myriad of new questions for the field of photoperiodism, one of which is: could photoperiodic responses have evolved before the emergence of circadian clocks? Cyanobacteria have faced daily and seasonal light–dark cycles since the group first evolved (Williams, 1993; Chiang & Broccoli, 2023). Given that circadian timers underlie the timing of day and night length in many organisms, and that there are 365× more selective cycles for days than for years in any given time interval, it would be logical to conclude that circadian rhythms evolved before PPTM. But is that necessarily true?

In principle, a photoperiodic response does not necessitate a circadian clock per se, but rather simply a mechanism to measure the duration of the day and/or the night. This mechanism could be an oscillator, or it could be something simpler, akin to an ‘hourglass’ timer, in which dawn or dusk could trigger the start of a particular process that leads to the accumulation of a deciding factor; for example, in long days, this deciding factor could be accumulated above a certain threshold and promote a long‐day response, but in short days it does not accumulate to that point, and the response is not promoted. By and large, photoperiodic responses in eukaryotes rely on circadian clocks as the photoperiodic time measurer (although in some cases, hourglass mechanisms have been proposed (Bradshaw *et al*., 1998, 2003)). Yet, this reliance does not signify that circadian clocks are necessary for the evolution of a photoperiodic response, or even that daily clocks evolved before photoperiodism.

In *S. elongatus*, we have studied the gene expression patterns that underlie/correlate with the photoperiodic response we observed. Photoperiod had a significant effect on gene expression, with *c*. 25% of the transcriptome differentially expressed between short and long days. Among those genes, we observed an interesting trend, in which genes related to daytime stressors (light, redox, or heat stress) are overexpressed in long days, while those associated with nighttime stressors (low energy, cold) are overexpressed in short days (Jabbur *et al*., 2024). This led us to postulate the possibility that cyanobacterial photoperiodism could have evolved before circadian clocks by hijacking more ancient stress responses, such as the stringent response (which is promoted when nutrients, e.g. amino acids/nitrogen, are low (Urwin *et al*., 2024)) and the SOS response (promoted by DNA damage (Baharoglu & Mazel, 2014)). As photosynthetic organisms, cyanobacteria face quite distinct stressors during the day and the night: sunlight can cause UV damage and redox stress, but its absence leads to metabolic stress and starvation. The relative duration and intensity of these two kinds of stressors expand and contract across the year. In summer days, photoautotrophic cyanobacteria might easily produce enough energy during the day to survive through the night, but at the same time, they are faced with brighter light (particularly UV) for considerably longer intervals of time. During winter days, UV stress is lower, but the cells must somehow survive longer intervals of dark starvation while having a shorter interval in which sunlight is available to produce energy. Survival and optimal reproduction of early (cyano)bacterial cells could have been a balancing act between differentially promoting certain stress responses during the day and inhibiting these responses at night.

The nighttime stressors are of particular interest: surviving starvation requires either a complete metabolic shutdown, a great reduction of the metabolic rate, or an excellent energy‐storage system. A very ancient cell is unlikely to have the latter, and a complete shutdown would not allow it to make adaptive use of the night, for example, by timing light‐disrupted processes such as DNA replication to happen during the night (Pittendrigh, 1993); on the other hand, the main mechanism for inhibiting DNA replication/cell division, the SOS response, relies on the evolutionarily ancient genes recA and lexA. recA and its homologues are found across all kingdoms and are proposed to have evolved from a single common ancestor before the divergence of bacteria and the lineage that would give rise to Archaea and Eukarya (Lin *et al*., 2006). The origins of lexA are less clear, although it is widely found across bacteria (Erill *et al*., 2007).

**Fig. 2:**
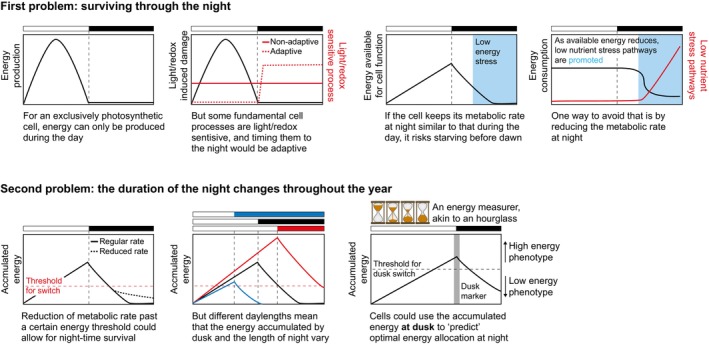
A possible pathway for the early evolution of photoperiodic responses, before the emergence of a circadian clock. Bars above each graph represent light–dark cycles, with the coloured bars (black, blue, or red) indicating darkness. When a plot has a double *y*‐axis, the black lines refer to the black‐labeled axis, and the red lines refer to the red‐labeled axis. See text for details.

If a cell is to both perform light‐disrupted processes at night and survive (i.e. not use up all of its energy reserves), it needs to carefully control its metabolic rate (Fig. 2, upper panels). Initially, a photosynthetic cell trying to survive the night might modulate its metabolism as a simple and direct response to current energy levels, such that when they fall below a certain threshold, metabolism is greatly reduced or shut off. This could be achieved by modulation of the ancient low‐nutrient response, which in cyanobacteria is promoted by darkness and inhibited by light (Puszynska & O'Shea, 2017), and is necessary for optimal growth and viability under light–dark cycles (Hood *et al*., 2016).

While such a mechanism could potentially work for nighttime survival, a *fixed* threshold after which a reduction of metabolic rate would be induced would be less optimal in circumstances in which cells face changes in day length. During long days, cells might be able to store enough energy during the day that they would be able to perform light‐sensitive processes at a regular rate throughout the night without ever running out of energy, but during short days, a much higher threshold might be needed in order to avoid starvation (Fig. 2, lower panels). Without a timing mechanism, a cell cannot know at the onset of dusk how long that darkness will last, but it could use the accumulated energy during the day as an approximate proxy. By tying an energy‐measuring mechanism with a way of marking dusk, cells could modulate their metabolic rate from the onset of the night and better utilize the short nights of summer while still surviving the long nights of winter. The mechanisms involved in this hypothetical scenario do not have to be extant proteins, but it is tempting to suggest that KaiC could be part of an energy‐measuring mechanism (similar to that proposed by Hut & Beersma, 2011), while KaiB could have signaled the onset of dark with its structural fold switching (Tseng *et al*., 2017). Of note, the KaiB fold switch is found in *Rhodobacter sphaeroides* (Wayment‐Steele *et al*., 2024), a bacterium that only has KaiB and KaiC and appears to have an hourglass rather than a circadian clock (Pitsawong *et al*., 2023).

This mechanism would effectively create an ‘energy‐measurer’ akin to an hourglass, which, by virtue of energy being provided by sunlight, can also double as an approximative photoperiodic time‐measurer (approximative because it does not measure the length of the day and the night per se, but rather uses energy as a proxy for them). Such a system would allow for adaptive usage of the night and nighttime survival, and would also potentially allow for the creation of mechanisms to anticipate recurring stressors associated with particular day lengths. The cold temperatures of winter or high UV levels of summer, for example, could be anticipated by evolving interactions between the low‐nutrient response and the cold/heat or DNA repair responses (interactions between the stringent response and cold adaptation/SOS response have been reported in other species (e.g. Strugeon *et al*., 2016; Wood *et al*., 2019)).

However, such a measurement would have a considerable degree of error – as it measures energy, not day length; a cloudy summer day could result in less photosynthesis and be interpreted the same as a short day, and a spell of hot weather during fall or winter could increase photosynthetic rates (Mackey *et al*., 2013) and be seen as a long day. Both could lead to an adaptive response when it comes to nighttime metabolic rates, but would be less adaptive for regulating cold/heat/DNA repair responses. This error‐proneness could create selective pressure toward a proper oscillatory system, which would no longer simply measure energy, but rather have its own internal metabolic rhythm that it adjusts to the daily cycle of energy production to function as a true photoperiodic time‐measurer. Initially, the system driving this internal rhythm could have been an oscillator simpler than a circadian clock; for example, a damped oscillator. Fluctuations in temperature/light intensity and unexpected spells of warm or cold weather could also lead to selection toward a counter mechanism (which can, for example, differentiate proper shortening of days from cold spells) and temperature compensation. Of note, one bacterium that does not possess a circadian clock but rather has a more simple damped oscillator still displays some degree of temperature compensation (Ma *et al*., 2016), but another species does not (Pitsawong *et al*., 2023).

It has been argued before (Pittendrigh, 1993) that circadian oscillators could have evolved from an ‘Escape from Light’ mechanism, in which the deleterious effects of light would select for the temporal segregation of DNA replication to the nighttime. However, under constant photoperiods, this kind of segregation could be well achieved with a simpler timing mechanism, like an hourglass (Johnson *et al*., 2017), and indeed mathematical models (Troein *et al*., 2009) suggest that selection for self‐sustainability (one of the hallmark definitions of circadian clocks) only occurs when there is variation in day length and other environmental variables. Hut and Beersma also hypothesized that KaiC could have evolved as an energy storage mechanism, allowing for maintenance of a constant ATP concentration through slow release of ATP throughout the night, while KaiB signaled the onset of darkness and KaiA turned the system into a self‐sustained oscillator that would allow for differential regulation of the phosphorylation rate of KaiC (Hut & Beersma, 2011). Here, we suggest that an energy‐measuring system (perhaps through KaiC), initially evolved to allow for survival through the night, served as the basis of a crude photoperiodic time‐measurer, and this energy‐measuring system could have eventually become the basis of a circadian clock. While such a proposition cannot be tested experimentally, it is possible to test whether other bacteria that do not appear to have self‐sustained circadian clocks (like *Rhodobacter* or *Rhodopseudomonas*) are capable of photoperiodic responses, and from these studies, one can begin to trace the evolutionary origins of photoperiodism.

Everything that we lay out here is purely speculative. We do not mean to imply that the current evidence *indicates beyond doubt* that photoperiodism predates circadian clocks. But we believe that, currently, it is impossible to say that circadian clocks are evolutionarily more ancient than photoperiodic responses. When photoperiodism was thought to be a phenomenon exclusive to eukaryotes, asking such questions as ‘What came first, photoperiodism or circadian clocks?’ would seem preposterous. The molecular machinery underlying (the oldest) circadian clocks is, after all, almost as ancient as the oldest lineage still in existence. But if photoperiodism is also present in this lineage, and if it is shown to be not merely found in a single species but rather widespread, then perhaps our ‘clock for all seasons’ (Pittendrigh & Daan, 1976) could actually be the result of all seasons coming together to make a clock.

## Competing interests

None declared.

## Disclaimer

The New Phytologist Foundation remains neutral with regard to jurisdictional claims in maps and in any institutional affiliations.
